# Crinophagy in Pancreatic Beta Cells: From Insulin Granule Turnover to Diabetes Pathogenesis

**DOI:** 10.3390/pathophysiology33030045

**Published:** 2026-07-03

**Authors:** Muralidharan Mani, Thomas F. J. Martin

**Affiliations:** Department of Biochemistry, University of Wisconsin-Madison, Madison, WI 53706, USA; tfmartin@wisc.edu

**Keywords:** crinophagy, insulin secretory granules, pancreatic beta cells, type 1 diabetes, type 2 diabetes, hybrid insulin peptides, crinosomes, SINGD, peripheral–thymic mismatch, cathepsin D

## Abstract

Pancreatic β-cells maintain glucose homeostasis through tightly regulated insulin biosynthesis, storage, and secretion. To prevent pathological accumulation of excess or aging secretory granules (SGs), β-cells use crinophagy, a selective lysosomal degradation pathway in which mature insulin-containing granules fuse directly with lysosomes to form hybrid organelles termed crinosomes. Crinophagy was historically considered a simple mechanism for discarding obsolete, aged SGs. The acidic, protease-rich environment of crinosomes is proposed to generate unconventional insulin-derived epitopes through cathepsin-mediated proteolysis and transpeptidation reactions. These cryptic epitopes, which include hybrid insulin peptides (HIPs) resulting from the covalent fusion of insulin fragments with peptides from co-resident granule proteins, are largely absent from the thymic epitope repertoire. This creates a “peripheral–thymic mismatch” that allows autoreactive CD4+ T cells to escape central tolerance, ultimately driving β-cell destruction in type 1 diabetes (T1D). Recent studies demonstrate that pharmacological or genetic inhibition of crinophagy reduces crinosome abundance, narrows the pathogenic epitope repertoire, and delays the onset of diabetes in preclinical models. In type 2 diabetes (T2D), a related pathway termed stress-induced nascent granule degradation (SINGD) diverts newly synthesized insulin granules to lysosomes under glucolipotoxic conditions, contributing to insulin depletion and progressive β-cell failure. This review summarizes the current understanding of the molecular mechanisms behind crinophagy. It discusses its two main functions: maintaining physiological quality control and generating pathological antigens. Additionally, the review explores how crinophagy interacts with other cellular stress pathways and highlights new therapeutic strategies aimed at targeting this process to protect pancreatic β-cell function and potentially prevent or delay diabetes.

## 1. Introduction

Pancreatic islets of Langerhans are central regulators of systemic metabolic homeostasis, coordinating the transition between fasting and feeding through precisely regulated hormone secretion [1]. Among islet cell types, β-cells are uniquely specialized for insulin production, devoting an extraordinary fraction of their biosynthetic capacity to this single hormone. In response to elevations in blood glucose, β-cells rapidly release insulin from secretory granules (SGs) to promote glucose uptake in peripheral tissues, whereas during fasting, insulin secretion is actively suppressed to prevent hypoglycemia, even as basal biosynthesis continues to replenish intracellular stores [2]. This intrinsic mismatch between continuous insulin production and episodic secretion necessitates robust mechanisms that control the size, composition, and functional competence of the granule pool [3]. At steady state, a single human β-cell contains approximately ten thousand insulin granules (mouse, 13,000), far exceeding the number released during any individual secretory burst. While this reserve enables rapid metabolic responsiveness, prolonged granule residence increases the risk of oxidative damage, protein misfolding, and loss of fusion competence, making unchecked accumulation metabolically costly and potentially deleterious. To preserve SG homeostasis, β-cells deploy multiple lysosomal degradation pathways that selectively remove supernumerary, aged, or dysfunctional granules, chief among them crinophagy, the direct fusion of secretory granules with lysosomes to form hybrid degradative organelles termed crinosomes [4,5,6].

Although originally described as a morphological phenomenon, crinophagy is now recognized as a dynamic regulatory process whose functional impact is shaped by the broader architectural and signaling context of the islet [7,8]. Mouse and human pancreatic islets share core organizational principles, including dense vascularization, coordinated hormone output, and intercellular coupling, yet differ markedly in cellular composition, three-dimensional architecture, and network behavior. Rodent islets typically exhibit a β-cell-enriched core with highly synchronized activity supported by gap-junction coupling and polarized organization around capillaries, whereas human islets contain a lower proportion of β-cells, greater α-cell abundance, and more heterogeneous cellular arrangements that vary across individuals and pancreatic regions [7,8]. These architectural differences likely influence how paracrine signals, blood flow, and calcium dynamics are integrated at the level of the β-cell, contributing to species-specific differences in stimulus–secretion coupling and stress responses [8]. Together, these considerations underscore why rodent models capture fundamental principles of β-cell biology, while quantitative features of SG turnover, secretory dynamics, and adaptive stress responses often require validation in human islets or human stem-cell-derived β-like systems.

## 2. Historical Perspective: From Morphological Curiosity to Molecular Mechanism

Crinophagy was first documented in the 1960s through electron microscopy studies of endocrine tissues, including pituitary [6], thyroid [9] and pancreatic islets [10,11]. Smith and Farquhar described lysosome-like organelles containing partially degraded secretory material, and Christian de Duve subsequently introduced the term crinophagy to denote direct SG–lysosome fusion. Early ultrastructural analyses identified crinosomes as electron-dense bodies containing remnants of SGs embedded within hydrolase-rich matrices characteristic of lysosomes. Parallel observations in liver cells by Ahlberg and Glaumann (1985–1987) established crinophagy as a conserved degradative mechanism across secretory cell types [12,13,14].

**Figure 1:**
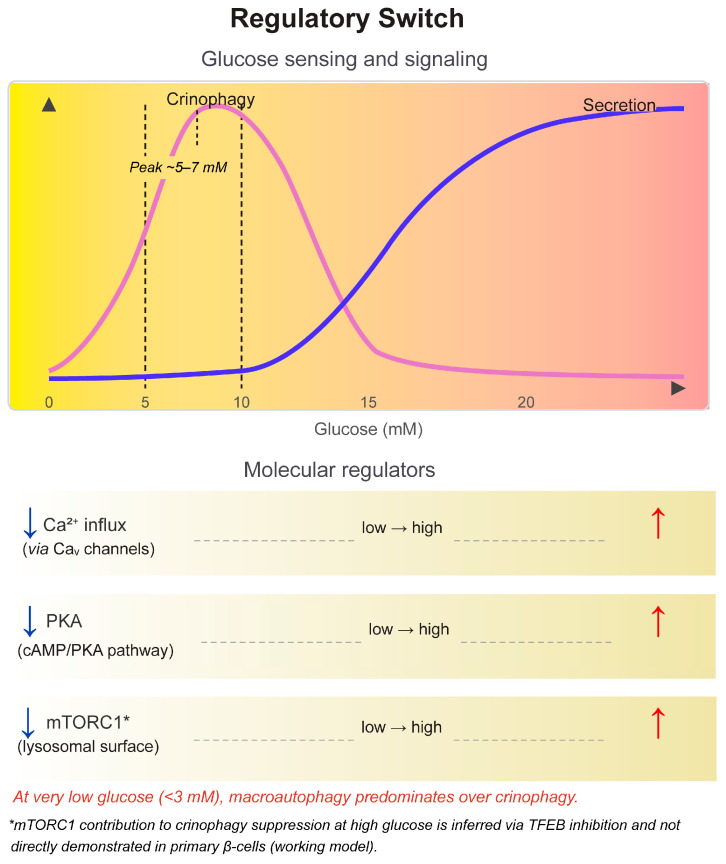
Glucose-dependent regulation of intracellular insulin degradation and lysosomal subpopulations in pancreatic β-cells. Schematic representation of the relationship between extracellular glucose concentration, intracellular insulin degradation, and lysosomal composition in β-cells. At low glucose (3.3 mM), intracellular degradation is elevated and the lysosomal compartment is enriched in insulin-negative secondary lysosomes, consistent with increased autophagic activity. At physiological glucose (5.5 mM), crinophagic degradation of insulin granules is maximal, reflecting accumulation of newly synthesized granules that exceed secretory demand. At high glucose (28 mM), insulin degradation is minimized as granules are preferentially released by exocytosis, resulting in a predominance of primary lysosomes and reduced crinophagic activity. The lower panel outlines key molecular regulators, where blue down arrows and red up arrows indicate decreased and increased pathway activity. Adapted from morphometric analyses of mouse islets reported by Pasquier et al. and Borg & Schnell [4,18].

For several decades, crinophagy was viewed primarily as an energy-efficient disposal pathway that eliminated excess hormone during periods of low secretory demand [15]. In pancreatic β-cells, where insulin-containing SGs have an estimated half-life of approximately 3–5 days, crinophagy was considered essential for basal turnover when biosynthesis exceeded secretion. SG degradation increases under intermediate glucose conditions [4] and decreases when glucose strongly stimulates exocytosis [16,17], consistent with a reciprocal relationship between secretion and degradation (Figure 1).

Genetic models further supported a homeostatic role. In settings where exocytosis is impaired, such as in Rab3A deficiency, SGs initially accumulate but are subsequently cleared through enhanced lysosomal degradation, including crinophagy- and autophagy-related pathways [5,19]. Similarly, genetic perturbations of IA-2β [20], Rab27a, Munc13-4, and VAMP2 result in impaired crinophagy and an increase in the SG count [21]. These observations established SG turnover as a compensatory mechanism preventing cytoplasmic overcrowding.

More recently, the conceptual framework surrounding crinophagy has shifted. Rather than functioning solely as a degradative endpoint, crinosomes have emerged as immunologically active organelles that generate hybrid insulin peptides that stimulate autoreactive CD4^+^ T cells in type 1 diabetes. Experimental attenuation of crinophagy in autoimmune-prone models reduces epitope presentation and delays the onset of diabetes, positioning crinosomes at the intersection of secretory biology and autoimmunity [22].

In parallel with these emerging insights into the expanded roles of crinophagy, broader studies of lysosomal pathways in β-cells have underscored the importance of macroautophagy in mitigating endoplasmic reticulum stress, oxidative damage and nutrient overload. β-cell-specific deletion of autophagy genes, such as Atg7 [23] or Atg5 [24], results in glucose intolerance, accumulation of ubiquitinated protein aggregates, and progressive β-cell dysfunction. Conversely, a mutant knock-in of the autophagy initiation protein beclin on a high-fat diet revealed that enhanced autophagy leads to impaired glucose tolerance despite improved insulin sensitivity [25]. However, macroautophagy and crinophagy are mechanistically and functionally distinct pathways, and their contributions to diabetes pathogenesis are not interchangeable. Whereas excessive crinophagy may amplify autoantigen generation in type 1 diabetes [22], dysregulated lysosomal routing of nascent SGs contributes to insulin depletion in type 2 diabetes [18]. Together, these findings redefine SG degradation as a central node in β-cell physiology and disease.

## 3. Molecular Players Involved in Crinophagy

### 3.1. Modes of Secretory Granule (SG) Entry into Lysosomes

**Figure 2:**
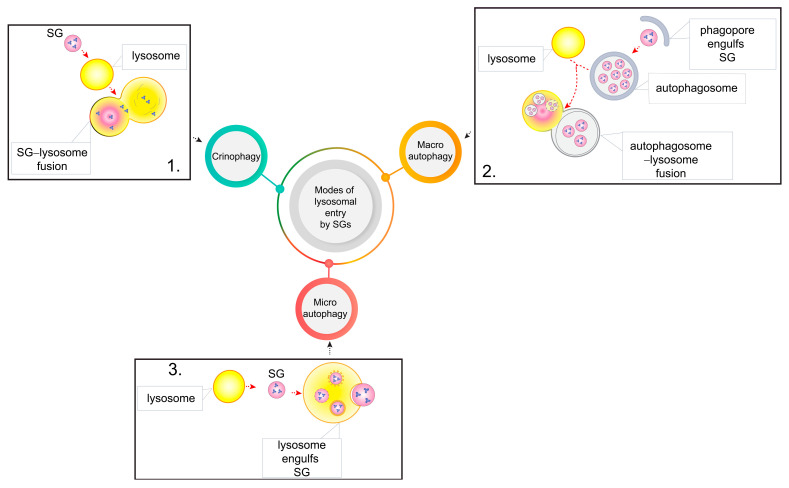
SGs are transported to the lysosomal compartment via diverse pathways. Schematic illustration of the primary pathways through which SGs enter the lysosomal degradative network. SGs can be targeted to lysosomes via macroautophagy (2, orange), in which SGs are sequestered within double-membrane autophagosomes before lysosomal fusion; microautophagy (3, red), which involves direct invagination of the lysosomal membrane to engulf SG material; or crinophagy (1, teal), a specialized pathway characterized by the direct fusion of mature SGs with lysosomes to form hybrid degradative compartments (crinosomes). These routes are not mutually exclusive and may be differentially activated depending on SG age, functional competence, metabolic state, and cellular stress. Collectively, these mechanisms provide β-cells with flexible strategies to regulate SG turnover and maintain intracellular SG homeostasis.

Pancreatic β-cells employ multiple lysosomal degradation pathways to maintain insulin granule homeostasis [5]. These pathways differ fundamentally in how SGs are delivered into the lysosomal lumen and in the handling of SG membrane components (selective retrieval/recycling versus bulk degradation) (Figure 2). Intracellular insulin-SG digestion can be attributed to their entry into lysosomal compartments. Three mechanisms allow SG entry into lysosomes: 1. crinophagy, 2. macroautophagy, and 3. microautophagy. Crinophagy involves the fusion of an SG with a lysosome, known as a “crinophagic body/crinosome,” resulting in the degradation of the insulin SGs.

Crinophagy proceeds through direct fusion of a single-membrane insulin SG with the single limiting membrane of lysosomes, generating a hybrid degradative organelle termed a crinophagic body/crinosome [12,26]. This single-membrane fusion delivers SG cargo, including insulin, C-peptide, and prohormone intermediates, directly into the lysosomal lumen for selective degradation of SG contents while preserving SG membrane components for potential recycling [4,5,6,18].

Macroautophagy, in contrast, involves capture of autophagic cargoes, including insulin, within the formation of a double-membrane structure called an “autophagosome” around the insulin SGs. The autophagosome subsequently docks with a lysosome, followed by fusion. Upon fusion of the autophagosome with the lysosome, the outer autophagosomal membrane merges with the lysosomal membrane, whereas the inner membrane and the enclosed SG are delivered intact into the lysosomal lumen. As a result, both SG cargo and SG membrane components are degraded with the autolysosome. This process is also called vesicophagy [25]. Macroautophagy requires the canonical autophagy machinery, including ATG proteins, the ULK1 complex [27], and LC3 lipidation [28].

Microautophagy represents a third, topologically distinct mechanism in which the limiting membrane of a late endosome or lysosome directly invaginates or protrudes to engulf SG material. In this pathway, insulin SGs are internalized into the lysosomal lumen through membrane remodeling events driven by the compartment itself. Because SG cargo is enveloped by the limiting membrane during internalization, both SG contents and the SG membrane are degraded within the resulting multivesicular body [29].

Thus, the fundamental distinction among these pathways lies in membrane topology and cargo engulfment. Crinophagy involves direct single-membrane fusion with selective luminal secretory cargo degradation and potential membrane conservation, whereas macroautophagy and microautophagy result in complete degradation of both SG cargo and membrane through membrane-enclosed delivery into the degradative lumen.

**Figure 3:**
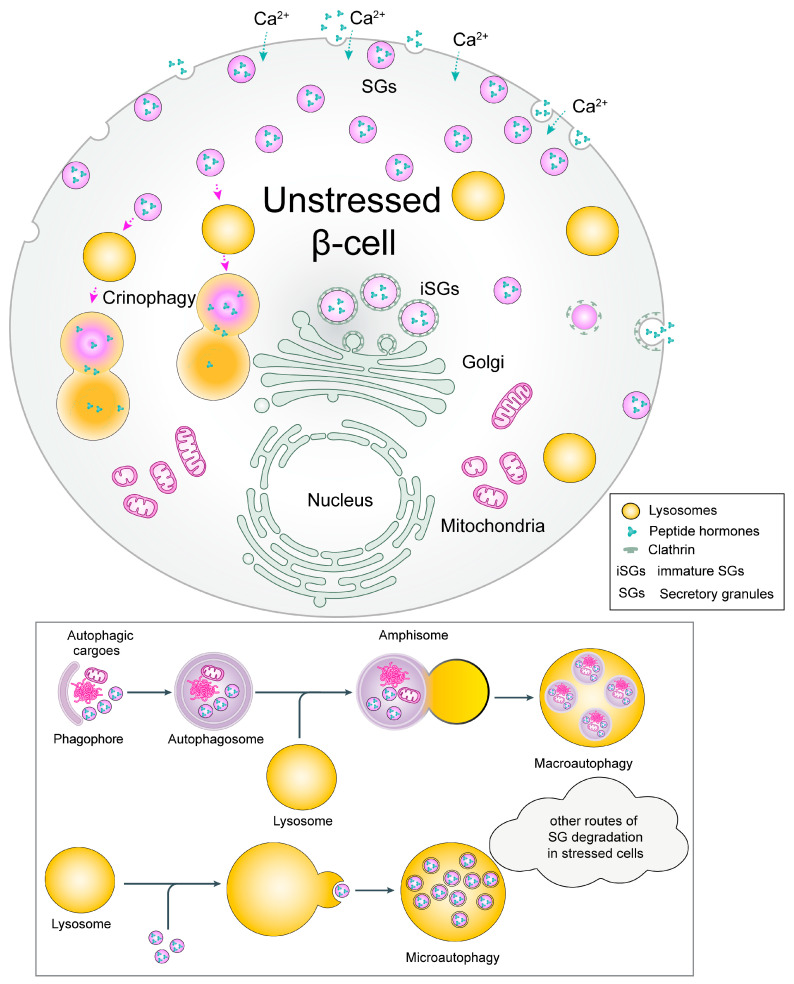
Lysosome-mediated pathways regulating SG turnover in pancreatic β-cells. (**Top**) Schematic overview of insulin SG biogenesis, trafficking, secretion, and basal degradation. Prohormone is packaged into Immature secretory granules (iSGs) at the trans-Golgi network, where peptide processing and SG maturation occur before SGs are transported toward the plasma membrane. Glucose-stimulated Ca^2+^ influx triggers exocytosis of a subset of SGs, releasing insulin. To maintain SG homeostasis, excess, aged, or dysfunctional SGs are selectively removed via crinophagy, a pathway involving direct fusion of mature SGs with lysosomes to form crinosomes. (**Bottom**) In addition to crinophagy, SGs can be degraded by alternative lysosomal routes. During macroautophagy, SGs are engulfed by an autophagosome that subsequently fuses with lysosomes. During microautophagy, the lysosomal membrane directly engulfs SGs. Together, these dynamic pathways operate alongside ongoing granule biosynthesis and regulated secretion, ensuring balance between insulin production, storage, and turnover under fluctuating metabolic demands. In the schematic, dashed teal arrows indicate Ca^2+^ influx and exocytotic release; dashed pink arrows highlight the targeting and fusion events of crinophagy and solid black arrows denote the sequential progression of the macro- and microautophagy pathways.

Notably, crinophagy operates independently of the canonical autophagy machinery and may represent a major pathway for homeostatic insulin SG turnover under non-stressed conditions [5,21]. This process enables selective degradation of the SG content, presumably leaving the SG membrane intact and enabling recycling (Figure 3) (Table 1).

### 3.2. Molecular Architecture of Crinophagy in Pancreatic β-Cells

Recent advances have revealed the molecular machinery governing insulin SG–lysosome fusion in mammalian β-cells, identifying distinct protein complexes on SG and lysosomal membranes that coordinate this selective degradation pathway.

The molecular machinery governing SG–lysosome fusion has been characterized in detail in the BON neuroendocrine cell model [21]. BON cells are a human neuroendocrine cell line that exhibits characteristics of immature pancreatic β-cell progenitors [30]. Like β-cells, BON cells contain numerous large SGs that store secretory peptides, including chromogranins A-C and neuotensin, and biogenic amines such as serotonin (5-HT), which are released via Ca^2+^-triggered exocytosis [31,32]. Chromogranins are core SG components shared across neural and endocrine cell types [33], making BON cells a tractable model for dissecting the molecular requirements of SG–lysosome fusion. While these findings provide a compelling framework for understanding crinophagy, their applicability to pancreatic β-cells has been only partially validated and should be considered a working model subject to revision as β-cell-specific studies emerge (Figure 3).

### 3.3. SG-Associated Machinery

#### 3.3.1. RAB27A: Possible Master Regulator of SG Fate

RAB27A, a small GTPase enriched on insulin SGs, has emerged as an important contributor to pancreatic β-cell homeostasis, linking intracellular SG turnover to metabolic dysfunction [21,34]. RAB27A serves dual roles in both SG exocytosis and crinophagy, with its activity state and effector interactions determining whether granules undergo secretion or lysosomal degradation [21]. Studies in BON cells suggest that in its GTP-bound active form, RAB27A recruits effector proteins to the SG membrane, including Munc13-4, which directs granules toward lysosomal fusion [21,35,36]. Based on these observations primarily in BON and INS-1 cell models, we propose a working model in which the ratio of RAB27A to RAB3A activity on the SG functions as a molecular “timer” that marks SG age: newly formed SGs enriched in RAB3A are preferentially routed to the plasma membrane for exocytosis [37], whereas older SGs with increased RAB27A activity become susceptible to crinophagic degradation [21]. Supporting this model, RAB3A-deficient mice exhibit increased insulin SG degradation via crinophagy and other lysosomal pathways, suggesting that loss of the RAB3A “youth marker” redirects SGs toward degradative fates. Conversely, expression of constitutively active RAB27A enhances crinophagy, whereas RAB27A knockdown impairs SG–lysosome fusion and reduces crinophagic flux [21]. Collectively, these findings are consistent with RAB27A functioning as a key node integrating SG age, positioning, and degradative targeting, though direct real-time visualization of RAB27A/RAB3A exchange dynamics on individual granules in human β-cells remains an important future goal.

As characterized in BON cells, crinophagy is mediated by a dedicated fusion machinery molecularly distinct from both macroautophagy and endolysosomal trafficking pathways [21]. Whether this precise machinery operates in pancreatic β-cells has not been directly tested, though the conservation of key components, including RAB27A and Munc13-4, across secretory cell types supports its broader relevance.

#### 3.3.2. Molecular Machinery of Insulin SG–Lysosome Fusion

The crinophagy fusion machinery centers on three functionally coupled components: an SG-associated priming factor, a dedicated Soluble NSF Attachment Protein Receptor (SNARE) complex, and a lysosomal tethering apparatus. Central among these is Munc13-4 (*unc13d*), a multi-domain Ca^2+^-sensing priming factor that couples SG recognition and tethering to membrane fusion [21,35]. Because this molecular framework was primarily characterized in BON neuroendocrine cells, the following sections describe each component in turn with explicit notation of where evidence is derived from BON cells versus pancreatic β-cells. Munc13-4 localizes to mature insulin granules through direct interaction with GTP-bound RAB27A, positioning it at the SG surface before lysosomal engagement [21]. Structurally, Munc13-4 contains tandem Ca^2+^-binding C2 domains, a central MHD domain that promotes SNARE complex assembly, and a C-terminal C2 domain implicated in membrane interactions [38,39]. This domain architecture resembles that of Munc13 family members that mediate regulated exocytosis, suggesting that a conserved priming function may be adapted for heterotypic organelle fusion [35].

In our model, Munc13-4 orchestrates SG–lysosome fusion through a series of sequential steps. Initial docking is mediated by interactions between SG-bound Munc13-4 and lysosome-associated tethering machinery, followed by Ca^2+^-dependent priming that enables SNARE-mediated membrane merger. Importantly, Ca^2+^ binding is dispensable for SG–lysosome docking but required for progression to fusion, indicating that crinophagy is regulated at a post-tethering checkpoint [21].

This model, established in BON cells [21], likely reflects a general principle of Ca^2+^-gated SG–lysosome fusion in secretory cells, though whether Munc13-4 is the obligate priming factor in pancreatic β-cells, or whether a related Munc homology protein could substitute, remains to be determined. The survival of Munc13-4 knockout mice under unstressed conditions, despite Munc13-4’s proposed requirement for crinophagy, raises the possibility of functional redundancy in vivo.

#### 3.3.3. SNARE Specificity Defines Crinophagy as a Distinct Fusion Pathway

In endocrine cells, the balance between regulated secretion and degradation of SGs is tightly controlled by SNARE-dependent trafficking pathways. Specific pairing between vesicular and target membrane SNARE directs SG maturation. Among vesicular R-SNAREs, VAMP2 and VAMP4 have emerged as key determinants in SG maturation [40,41,42]. In the BON neuroendocrine model, the SG–lysosome involves a specific SNARE complex comprising the SG-localized R-SNARE VAMP2 and the lysosomal Q-SNAREs STX7, STX8, and VTI1B. Disruption of any component of this complex impairs crinophagic flux, whereas SNAREs required for autophagosome–lysosome fusion (such as STX17, YKT6 or SNAP29) are dispensable [21]. This molecular specificity underscores that crinophagy is mechanistically distinct from canonical autophagy-related fusion events. Whether this exact SNARE combination operates in pancreatic β-cells has not been directly demonstrated. This distinction matters: exocytosis in β-cells has been attributed to multiple SNARE combinations depending on context, and SNARE substitutions within fusion pathways are documented across cell types. The BON cell SNARE assignment thus defines a molecular framework for crinophagy that may be subject to cell-type-specific variation.

In contrast, the acquisition of VAMP2 marks SGs that enter the mature releasable pool and subsequently undergo constitutive crinophagy as they age. This VAMP4-to-VAMP2 transition provides a molecular mechanism by which β-cells distinguish nascent from mature SGs and route them to distinct fates.

**Figure 4:**
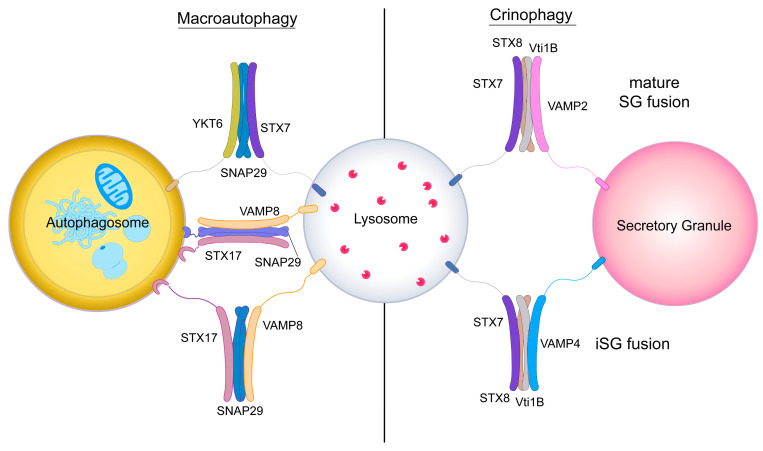
Distinct SNARE machineries mediate macroautophagy and crinophagy. A schematic comparison of the membrane fusion machinery governing autophagosome–lysosome fusion (macroautophagy) [43,44,45] and SG–lysosome fusion (crinophagy) [21]. In macroautophagy (left), double-membraned autophagosomes fuse with lysosomes through a canonical SNARE complex composed of Syntaxin 17 (STX17), SNAP29, and VAMP8 [45], with additional contributions from YKT6-SNAP29-STX7 complexes. In crinophagy (right), secretory granules undergo direct fusion with lysosomes via distinct SNARE assemblies that depend on the maturation state of the granules. Mature SGs predominantly engage a STX7-STX8-VTI1B-VAMP2 [21] complex, whereas immature secretory granules (iSGs) utilize a related but distinct SNARE configuration involving STX7-STX8-VTI1B-VAMP4 [46]. This divergence in SNARE usage enforces pathway specificity, mechanistically distinguishing crinophagy from canonical macroautophagy while enabling selective lysosomal entry of SGs throughout their lifecycle.

Together, these findings support a dual-pathway framework: mature SG crinophagy, characterized in the BON cell model via the VAMP2 SNARE complex and dedicated tethering apparatus, operates in parallel with VAMP4-mediated SINGD targeting iSGs. While the precise conservation of the mature crinophagy fusion apparatus in pancreatic β-cells remains to be directly established, this point of convergence between the BON cell framework and the β-cell literature explains how secretory cells integrate SG quality control with metabolic adaptation. This integrated molecular architecture provides a mechanistic foundation for understanding how dysregulated SG degradation contributes to diabetes (Figure 4). Importantly, while the core crinophagy molecular machinery, including Munc13-4, PLEKHM1, the HOPS complex, and the VAMP2-containing SNARE complex, has been rigorously characterized in BON neuroendocrine cells, its direct functional equivalent has not yet been demonstrated in primary human β-cells.

#### 3.3.4. Tethering Machinery at the SG–Lysosome Interface

SG capture by lysosomes is facilitated by the HOPS (homotypic fusion and protein sorting) complex, a conserved multisubunit tethering factor that functions broadly in lysosomal fusion—a mechanism primarily characterized in fibroblasts and BON cells. In fibroblast cells, HOPS is recruited to lysosomes via its adaptor PLEKHM1, which together helps establish lysosomal identity and competence for fusion [47]. Consistent with this, both PLEKHM1 and HOPS have been implicated in crinophagy of glue granules in *Drosophila*, where they are required for fusion with lysosomal compartments [48].

In the BON neuroendocrine cell, Munc13-4 interacts with multiple HOPS subunits, suggesting that it may function as an SG-localized factor that couples SGs to lysosomal tethering machinery. This proposed Munc13-4-PLEKHM1-HOPS axis provides specificity to SG capture by ensuring selective engagement of lysosomes rather than indiscriminate fusion with other intracellular organelles [21]. Following the tethering process, the SM protein VPS33A within the HOPS complex stabilizes productive SNARE pairing, promoting efficient membrane fusion [49]. Whether an equivalent tethering mechanism operates in pancreatic β-cells remains unresolved, although HOPS-dependent lysosomal fusion is broadly conserved across secretory and non-secretory cell types [21,47,48].

In addition to the Munc13-4-PLEKHM1-HOPS tethering axis, a compositionally distinct tethering module has been proposed in rodent β-cell models. Zhou et al. demonstrated in MIN6 and INS-1 cells that the RAB7 effector RAB-interacting lysosomal protein (RILP) interacts with SG-associated RAB26 and lysosomal RAB7, bridging iSGs to the lysosomal compartment and promoting proinsulin degradation in a RAB7-dependent manner [50]. RILP expression is upregulated in islets from HFD-fed rats and db/db mice, and RILP loss of function increases proinsulin levels and enhances insulin secretion, suggesting pathophysiological relevance under diabetic conditions. However, RAB7 knockdown in MIN6 cells rescued RILP-induced proinsulin degradation [50], whereas RAB7 knockdown in BON cells did not significantly impair crinophagic flux, a divergence that likely reflects the absence of RAB7 from lysosomes in BON cells [21] and the unresolved subcellular identity of RAB7-positive structures in rodent β-cell lines, as RAB7 localization was not directly examined in MIN6 or INS-1 cells in the Zhou et al. study. Additionally, the RILP-RAB7 interaction was demonstrated by co-immunoprecipitation in non-endocrine cells rather than in β-cell lines [50], leaving open whether this interaction occurs at physiological expression levels in the specialized secretory environment of a β-cell. Whether the RAB26-RILP-RAB7 axis represents a β-cell-specific tethering pathway, a route selectively engaged under RILP overexpression conditions, or a late endosomal rather than strictly lysosomal targeting mechanism remains to be determined.

#### 3.3.5. Ca^2+^-Dependent Regulation of Crinophagy

A defining feature of crinophagy in BON cells is its requirement for Ca^2+^ at a post-tethering step. Munc13-4 functions as a Ca^2+^ sensor, with Ca^2+^ binding triggering conformational changes that promote SNARE assembly and membrane merger. Chelation of intracellular Ca^2+^ selectively abolishes SG–lysosome fusion without disrupting prior docking, demonstrating that Ca^2+^ is dispensable for tethering but strictly required for progression to the membrane merger event.

The spatial origin of the Ca^2+^ signal that triggers membrane merger remains unresolved. Pharmacological chelation experiments using fast (BAPTA) and slow (EGTA) Ca^2+^ buffers showed only partial preferential inhibition by BAPTA under some experimental conditions, which is consistent with, but does not prove, a locally restricted Ca^2+^ source at the SG–lysosome interface [21]. As a working hypothesis, the lysosomal cation channel TRPML1 represents a plausible candidate for providing this local Ca^2+^ signal, given its established role in driving membrane fusion at late endosomal and lysosomal compartments in other cellular contexts. However, whether TRPML1 participates in crinophagy specifically has not been directly tested, and this remains an open question that future studies using TRPML1-selective agonists (ML-SA1) and genetic knockdown in β-cell models could resolve.

#### 3.3.6. Integration with Metabolic and Inflammatory Signaling

**Figure 5:**
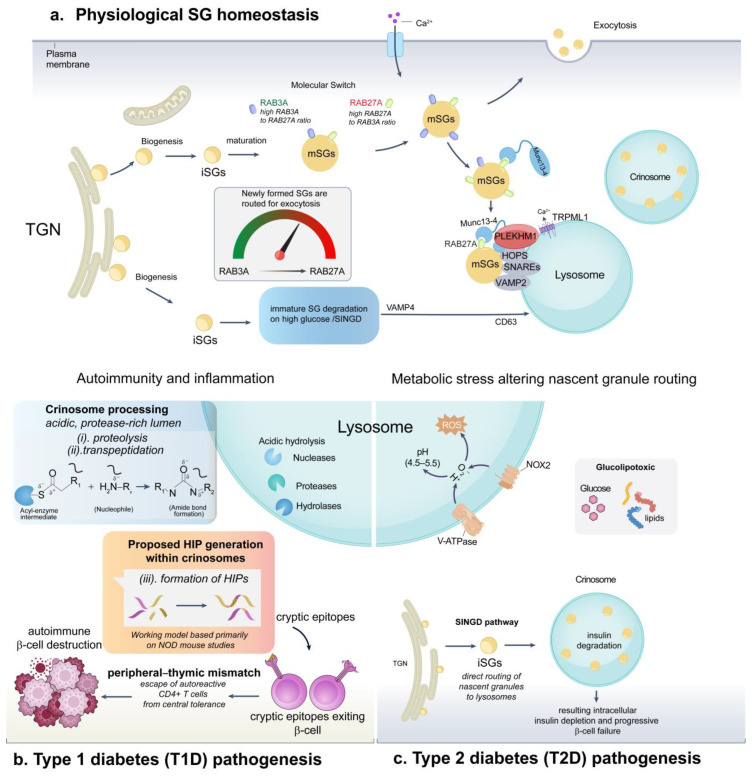
Physiological secretory granule homeostasis and its divergent contributions to type 1 and type 2 diabetes pathogenesis. (**a**) Physiological SG homeostasis. Insulin secretory granules are generated at the trans-Golgi network (TGN) as immature secretory granules (iSGs) and undergo maturation into mature secretory granules (mSGs). A proposed molecular switch governs SG fate as granules age: newly synthesized mSGs enriched with multiple RAB3A molecules (depicted by high RAB3A abundance on the granule surface) are preferentially routed to the plasma membrane for Ca^2+^-triggered exocytosis, whereas aging mSGs that progressively accumulate RAB27A (depicted by high RAB27A abundance on the granule surface) are redirected toward lysosomal degradation via crinophagy. This RAB3A-to-RAB27A transition on the SG surface functions as a proposed molecular timer of granule age, though this model has not been directly validated in primary human β-cells. Crinophagy proceeds through a dedicated fusion complex—comprising RAB27A-recruited Munc13-4, the lysosomal tethering factors PLEKHM1 and HOPS, and a VAMP2-containing SNARE complex, as characterized in the BON neuroendocrine cell model—mediating direct mSG–lysosome fusion to form hybrid degradative organelles termed crinosomes. The lysosomal cation channel TRPML1 is depicted on the lysosomal membrane as a candidate source of locally released Ca^2+^ that may contribute to the Ca^2+^-dependent membrane fusion step, although its direct involvement in crinophagy has not been experimentally demonstrated. Under glucolipotoxic or metabolic stress conditions, newly synthesized iSGs are diverted from the biosynthetic–secretory route toward lysosomes via VAMP4 and CD63, constituting stress-induced nascent granule degradation (SINGD). (**b**) Type 1 diabetes (T1D) pathogenesis. The central lysosome depicts the general biochemical environment shared by both degradation contexts: the acidic lumen (pH 4.5–5.5), maintained by V-ATPase-driven proton pumping and supported by NOX2-mediated ROS generation, is enriched in nucleases, proteases, and hydrolases. The proposed mechanisms suggest that within crinosomes, this protease-rich acidic environment supports cathepsin-mediated proteolysis and transpeptidation reactions, generating hybrid insulin peptides (HIPs)—cryptic neoepitopes arising from the covalent fusion of insulin or C-peptide fragments with peptides derived from co-granule proteins. These HIPs might exit the β-cell and, because they are largely absent from the thymic antigen repertoire, activate autoreactive CD4^+^ T cells through peripheral–thymic mismatch, ultimately driving autoimmune β-cell destruction in T1D. (**c**) Type 2 diabetes (T2D) pathogenesis. Under glucolipotoxic conditions, the SINGD pathway diverts newly synthesized iSGs from the TGN directly to lysosomes for degradation, bypassing the functional secretory pool. Sustained SINGD results in progressive intracellular insulin depletion and impaired glucose-stimulated insulin secretion, contributing to the β-cell failure characteristic of T2D.

Crinophagy is modulated by metabolic and inflammatory cues that influence lysosomal activity. Nutrient sensing through mTORC1 intersects with SG degradation pathways, particularly during SINGD, where amino acids released from lysosomal granule degradation activate mTORC1 and suppress macroautophagy [51]. Nitric oxide and prostaglandin signaling pathways provide tonic regulation of crinophagic flux, and inflammatory cytokines enhance granule degradation under stress conditions [11]. How these signals converge on the dedicated SG–lysosome fusion machinery (comprising RAB27A, Munc13-4, HOPS/PLEKHM1 and the VAMP2-STX7-STX8-VTI1B SNARE complex) as characterized in BON cells [21] or their β-cell counterparts remains an open question (Figure 5). Beyond acute inflammation regulation, crinophagic flux may also be governed by nutrient-sensing pathways that coordinate lysosomal capacity with cellular metabolic state, as discussed below.

#### 3.3.7. Potential Regulation of Crinophagy by the mTORC1-TFEB-AMPK Axis

Lysosomal degradation pathways are tightly integrated with cellular nutrient-sensing networks, suggesting that crinophagy is likely regulated by mechanisms that coordinate SG turnover with metabolic state. Among these, the mTORC1–TFEB–AMPK signaling axis serves as a central regulator of lysosomal function, autophagy, and cellular energy homeostasis.

mTORC1 is recruited to the lysosomal surface, where it integrates amino acid availability, growth factor signaling, and cellular metabolic status to promote anabolic processes [52]. A key downstream target of mTORC1 is the transcription factor TFEB, a master regulator of lysosomal biogenesis and autophagy-related gene expression. Under nutrient-replete conditions, active mTORC1 phosphorylates TFEB, retaining it in the cytoplasm and thereby suppressing transcriptional programs that expand lysosomal capacity. Conversely, nutrient deprivation or energetic stress reduces mTORC1 activity, allowing TFEB dephosphorylation, nuclear translocation, and activation of genes involved in lysosomal biogenesis, autophagic flux, and cellular clearance pathways [53,54].

AMP-activated protein kinase (AMPK) provides an additional layer of regulation by sensing cellular energy deficiency. Upon activation, AMPK antagonizes mTORC1 signaling and can further promote TFEB activation, thereby shifting cellular programs from anabolic growth toward catabolic recycling. Through this coordinated nutrient-sensing circuit, AMPK, mTORC1, and TFEB couple lysosomal function to cellular metabolic demands [54,55].

Direct evidence linking this regulatory axis to crinophagy remains limited. However, several observations suggest a plausible connection. In a clonal murine pancreatic α-cell line, pharmacological combined with genetic inhibition of mTORC1 promotes crinophagy-mediated degradation of glucagon-containing SGs independently of macroautophagy, demonstrating that mTORC1 can directly influence lysosomal granule turnover [56]. In parallel, TFEB-dependent lysosomal biogenesis is required for the efficient clearance of SGs in pancreatic acinar cells, where impaired TFEB activity leads to granule accumulation and cellular dysfunction [57]. Although these findings do not establish a direct role for TFEB in regulating crinophagic flux, they suggest that TFEB-mediated expansion of lysosomal capacity may create a permissive environment for crinophagy.

These observations support a working model in which nutrient abundance favors granule preservation through mTORC1 activation and TFEB suppression, whereas nutrient limitation or metabolic stress promotes lysosomal remodeling through AMPK activation, mTORC1 inhibition, and TFEB-dependent transcriptional programs. Whether this regulatory framework directly governs crinophagy in pancreatic β-cells remains unresolved. Future studies should determine whether mTORC1, TFEB, and AMPK regulate crinosome formation, secretory granule selection, or crinophagic flux under physiological and diabetogenic conditions. Such investigations may reveal how metabolic signals dynamically coordinate insulin granule turnover with β-cell functional demands and disease progression.

#### 3.3.8. Conservation and Divergence Across Systems

Comparative studies in *Drosophila* and mammalian secretory cells indicate that crinophagy operates under a conserved organizational framework. This framework includes selective recognition of SGs, Rab GTPase-dependent membrane identity, the recruitment of tethering complexes, and SNARE-mediated fusion with late endosomal or lysosomal compartments. However, the specific molecular components involved can differ across species and cell types.

#### 3.3.9. Molecular Framework of Crinophagy

Collectively, current evidence from the BON neuroendocrine cell model supports a working framework in which crinophagy is proposed to be mediated by a dedicated fusion complex. SG-bound RAB27A recruits Munc13-4, which serves as a Ca^2+^-dependent priming factor. Lysosomal engagement is facilitated by PLEKHM1 and the HOPS complex. While studies in *Drosophila* suggest that RAB7 anchors this tethering complex during crinophagy, the specific lysosomal Rab GTPase responsible for this function in the BON cell model or in pancreatic β-cells still needs to be identified. Together, these complexes capture SGs and stabilize the assembly of SNARE proteins. The fusion of these granules is carried out by a specific SNARE complex that consists of VAMP2, STX7, STX8, and VTI1B [21]. Whether this molecular architecture operates analogously in pancreatic β-cells remains to be established. Parallel pathways target iSGs via VAMP4-mediated SINGD, providing a maturation checkpoint that is characterized in INS-1 cells [46,51], thus offering an important point of convergence between the BON cell framework and the β-cell literature.

SINGD reflects a trafficking decision made early in the SG lifecycle, near the trans-Golgi network and iSG compartment. Under normal conditions, protein kinase D1 (PKD1) promotes forward trafficking and maturation of iSGs. However, under glucolipotoxic or nutrient stress, reduced PKD1 activity biases nascent granules toward lysosomal routing [18,51,58]. The lysosomal tetraspanin CD63 is required for this diversion of iSGs to lysosomes. Degradation of iSGs within lysosomes releases amino acids that activate mTORC1 at the lysosomal surface, which in turn suppresses macroautophagy initiation, thereby linking nascent granule degradation to broader crinophagy regulation through a lysosome-centered feedback mechanism [51].

Membrane fusion between iSGs and lysosomes is likely mediated by a dedicated SNARE complex. In INS-1 cell models, VAMP4 localized to iSGs assembles with lysosomal SNAREs STX7, STX8, and VTI1B to drive iSG–lysosome fusion, providing a molecular mechanism for SG delivery into the degradative pathway [46]. Whether this SNARE machinery operates specifically during stress-induced SINGD or more broadly during constitutive iSG quality control remains to be clarified, as does its relationship to upstream PKD1-CD63 signaling.

Sustained activation of SINGD results in degradation of newly synthesized granules before they enter the functional secretory pool, progressively reducing the number of releasable granules and impairing glucose-stimulated insulin secretion. Under chronic metabolic stress, this process may contribute to the decline in β-cell secretory capacity characteristic of type 2 diabetes [27,40,41,59].

This molecular architecture explains how β-cells integrate SG quality control with metabolic adaptation and provides a mechanistic foundation for understanding how dysregulated granule degradation contributes to diabetes.

## 4. Crinophagy in Diabetes Pathogenesis

Although both T1D and T2D involve lysosome-dependent degradation of insulin-containing granules, the underlying mechanisms, triggers, and pathological consequences are distinct and should not be conflated. In T1D, conventional crinophagy of mature secretory granules has been proposed to generate hybrid insulin peptides (HIPs) within the crinosome lumen through cathepsin-mediated transpeptidation [60]. These neoepitopes are thought to be absent from the thymic repertoire, enabling autoreactive CD4^+^ T cells to escape central tolerance and contribute to β-cell autoimmunity [59]. Thus, the relevant granule population is the mSG, the pathway is constitutive, and the trigger is the normal aging and turnover of granules within the secretory pathway.

By contrast, the principal lysosomal pathway implicated in T2D is stress-induced nascent granule degradation (SINGD), which targets newly synthesized immature secretory granules (iSGs) under conditions of glucolipotoxic stress. SINGD is driven by reduced PKD1 activity and CD63-dependent lysosomal targeting and results in depletion of the functional insulin secretory pool, impaired glucose-stimulated insulin secretion, and progressive β-cell dysfunction [18]. Consequently, the pathological outcome is metabolic failure rather than immune activation.

These distinctions in granule population targeted, molecular trigger, downstream consequence, and disease mechanism have important implications for therapeutic intervention. Whereas T1D-directed strategies seek to limit the generation of diabetogenic neoepitopes, T2D-directed approaches aim to preserve insulin granule abundance and secretory competence by preventing pathological degradation of nascent granules. Maintaining this distinction is therefore essential when considering the role of lysosomal granule degradation pathways in diabetes pathogenesis.

### 4.1. Type 1 Diabetes: The Crinosome as an Immunologically Active Organelle

A major conceptual shift in β-cell biology came from the observation that diabetogenic CD4^+^ T-cell clones (initially characterized in the NOD model) can recognize non-canonical insulin-derived epitopes, notably hybrid insulin peptides (HIPs). These neoepitopes arise when fragments of insulin or C-peptide become covalently linked to fragments derived from other SG constituents, including chromogranin A and islet amyloid polypeptide [61,62]. Subsequent detection of HIPs in islet material by mass spectrometry supported the conclusion that these species can be generated in vivo, rather than being experimental artifacts [63].

Mechanistic work has identified crinosomes, hybrid organelles formed by SG fusion of insulin SGs with lysosomes, as a probable site of HIP generation, though direct evidence that HIPs are produced within crinosomes specifically, rather than other lysosomal compartments, remains limited [64]. The acidic, protease-rich luminal environment of crinosomes supports both cathepsin-mediated proteolysis and transpeptidation-like reactions, enabling peptide fragments to become covalently ligated under conditions that differ from classical cytosolic antigen processing. Cathepsin D has been implicated in generating specific HIP species, while cathepsin L may degrade certain HIP precursors, suggesting that HIP abundance reflects a balance between anabolic and catabolic enzymatic activities within this compartment [22,65]. Additional cathepsins (for example, B, S, or V) may shape the substrate pool and thereby influence which hybrid products are formed, although their specific contributions remain incompletely resolved.

These findings introduce a broader immunological principle: β-cells can diversify the insulin-derived peptide landscape through post-translational peptide remodeling within lysosome-related compartments. This process expands the set of epitopes available for MHC class II presentation and may contribute to the breadth of autoreactive CD4^+^ T-cell responses observed in type 1 diabetes [66].

### 4.2. Evidence Linking Hybrid Insulin Peptides to Type 1 Diabetes: Mechanistic Insights and Translational Gaps

HIPs have emerged as important neoepitopes in type 1 diabetes (T1D), providing a potential mechanistic link between β-cell secretory granule processing and autoreactive CD4^+^ T-cell activation. Collectively, animal and human studies support the biological relevance of HIPs; however, the strength of evidence differs substantially between mechanistic studies in experimental models and translational observations in humans.

#### 4.2.1. Mechanistic Evidence from Experimental Models

Studies in the non-obese diabetic (NOD) mouse have established a compelling mechanistic framework linking HIP formation to autoimmune diabetes. Pathogenic CD4^+^ T-cell clones isolated from diabetic mice recognize HIPs generated through the covalent fusion of peptide fragments, and mass spectrometry has confirmed the presence of HIPs within pancreatic β-cells. These findings provide direct evidence that HIPs constitute naturally occurring neoantigens capable of driving diabetogenic T-cell responses [62,67].

Subsequent studies have begun to elucidate the molecular mechanisms underlying HIP biogenesis. Cathepsin D has been identified as a major contributor to the formation of disease-relevant HIPs within islet preparations, whereas genetic deficiency of cathepsin L alters HIP abundance in NOD islets, supporting a role for lysosomal proteases in HIP generation through transpeptidation reactions. Additional experimental work demonstrated that antigen-presenting cells (APCs) can generate HIPs under controlled conditions and present these neoepitopes to autoreactive CD4^+^ T cells, providing a plausible pathway for antigen processing and presentation [67].

Taken together, these studies establish a biologically coherent model in which proteolytic processing within β-cell secretory pathways generates HIPs that subsequently participate in pathogenic CD4^+^ T-cell responses. Importantly, the majority of mechanistic evidence supporting this pathway derives from murine systems and experimental models.

#### 4.2.2. Evidence from Human Studies

Human studies provide strong support for the immunological relevance of HIPs but offer less direct insight into their intracellular origin. These cells frequently display effector-memory and pro-inflammatory phenotypes and respond to naturally processed islet antigens, indicating that HIPs are recognized by the human autoimmune repertoire [68].

Mass spectrometry analyses have confirmed the presence of specific HIP sequences within human islets, and HIP-specific T-cell receptors have been recovered from pancreatic tissue, providing direct evidence that these neoepitopes exist in the human disease setting. Furthermore, longitudinal studies of individuals at risk for T1D have demonstrated that HIP-directed immune responses can precede clinical disease onset, suggesting a potential role in disease progression. Experimental studies also indicate that human APCs are capable of generating and presenting HIPs in vitro, raising the possibility that HIP formation may occur in multiple cellular compartments beyond β-cells [69].

Collectively, these findings establish that HIPs are present in human islets and are recognized by autoreactive CD4^+^ T cells. However, they do not directly define the intracellular compartment in which HIPs are generated nor demonstrate the complete sequence of events leading from β-cell granule processing to T-cell activation.

#### 4.2.3. Crinosomes as a Proposed Site of HIP Generation

Recent studies have proposed that crinosomes—hybrid organelles formed through fusion of insulin secretory granules with lysosomes—may provide a favorable microenvironment for HIP generation. The acidic lumen, high cathepsin activity, and abundance of peptide substrates within these compartments are consistent with conditions known to promote transpeptidation reactions. Cathepsin D and cathepsin L, both implicated in HIP formation, are enriched within lysosomal degradative pathways and therefore provide a mechanistic rationale for crinosome involvement [65].

Nevertheless, direct evidence demonstrating HIP generation within crinosomes remains limited. To date, no study has visualized HIP formation in crinosomes of primary human β-cells, nor has any investigation definitively established that crinophagy-derived HIPs are subsequently transferred to antigen-presenting cells and presented through MHC class II pathways in vivo. Consequently, the proposed crinosome → HIP → antigen presentation → CD4^+^ T-cell activation pathway should currently be regarded as a biologically plausible working model rather than an established mechanism.

#### 4.2.4. Outstanding Questions

A major challenge for the field is bridging the gap between mechanistic observations in experimental models and direct evidence in human disease. Future studies combining high-resolution imaging of human β-cell crinophagy, compartment-specific proteomics, mass spectrometric detection of HIPs within crinosomes, and ex vivo β-cell–APC–T-cell coculture systems will be required to determine whether crinophagy represents a dominant source of diabetogenic HIPs in vivo.

Thus, while experimental models provide strong mechanistic evidence linking secretory granule processing, HIP generation, and pathogenic CD4^+^ T-cell responses, human studies currently establish the presence and immunological relevance of HIPs without definitively demonstrating the complete crinosome-mediated pathway. This distinction is important when considering HIPs as both mechanistic drivers and potential therapeutic targets in T1D.

### 4.3. Peripheral–Thymic Mismatch and Tolerance

A key implication of HIP biology is that these hybrid epitopes are expected to be rare or absent during thymic selection, thereby increasing the likelihood that HIP-reactive T cells escape central deletion [67]. Thymic presentation of insulin relies on tissue-restricted antigen expression (including AIRE-driven mechanisms) and established antigen processing routes; however, the extent to which thymic antigen-presenting cells generate the same spectrum of lysosome-derived hybrid epitopes as β-cells remains uncertain [65]. A “peripheral–thymic mismatch” model therefore provides a compelling conceptual framework: T cells that are not tolerized to crinosome-derived neoepitopes can later be activated when these epitopes become available in the islet environment [22].

How crinosome-derived or HIP-containing peptides reach professional antigen-presenting cells (APCs) remains unresolved. At present, the most defensible interpretation is that several routes are plausible, but direct evidence for any single dominant pathway is still limited. One candidate route is lysosomal exocytosis, a process in which lysosomes fuse with the plasma membrane and release luminal contents extracellularly [70]; this mechanism is well established in cell biology, but its specific contribution to HIP export from β-cells has not been directly demonstrated. A second plausible route is extracellular vesicle release from the endolysosomal system, including exosomes derived from multivesicular bodies (MVBs). This possibility is supported indirectly by evidence that human and rat β-cells release autoantigen-containing exosomes that can be taken up by dendritic cells, although those studies did not specifically track HIPs or crinosome-derived cargo [71]. A third route is β-cell stress or cell death, which could expose or release lysosomal and SG-derived material for uptake and processing by resident or infiltrating APCs [65]. Collectively, these possibilities are consistent with the broader idea that inflammatory or stress-induced lysosomal remodeling in β-cells could expand the peripheral neoepitope pool and thereby amplify CD4^+^ T-cell activation, but the relative importance of each route remains to be defined experimentally.

### 4.4. Experimental Support and Therapeutic Rationale

Recent in vivo work supports the idea that modulating crinophagy/crinosome formation can reshape the islet epitope repertoire and attenuate diabetogenic T-cell responses in susceptible models [22]. Importantly, these studies suggest that partial, context-dependent suppression of crinophagy may reduce the generation or availability of unconventional epitopes without necessarily abolishing the insulin SG pool [72]. Together, these findings suggest that crinophagy may function not only as a homeostatic degradation pathway but also as a process with immune-amplifying consequences in susceptible settings. They motivate exploration of crinosome-targeted interventions as a disease-modifying strategy in early type 1 diabetes.

#### Potential Risks and Therapeutic Trade-Offs of Modulating Crinophagy

The therapeutic manipulation of crinophagy presents both opportunities and challenges. Although recent studies suggest that limiting crinophagy may reduce the generation of diabetogenic neoepitopes and delay the progression of autoimmune diabetes, crinophagy also serves essential physiological functions in secretory-cell homeostasis [22]. Consequently, sustained inhibition of this pathway could have unintended consequences for β-cell function and hormone secretion.

Under physiological conditions, crinophagy acts as a quality-control mechanism that selectively removes aged, damaged, or surplus secretory granules through lysosomal degradation. Early ultrastructural studies demonstrated dynamic interactions between lysosomes and insulin granules in pancreatic β-cells, with crinophagic activity varying according to glucose-dependent secretory demand [4]. More recent work in endocrine and secretory-cell systems has further shown that regulated lysosomal granule turnover contributes to maintenance of granule quality and secretory competence.

Experimental perturbation of lysosomal granule degradation pathways highlights the importance of balanced granule turnover. Excessive lysosomal consumption of insulin-containing compartments, as observed following β-cell-specific deletion of Atp6ap2, results in depletion of intracellular insulin stores and impaired glucose homeostasis [73]. Similarly, enhanced lysosomal degradation of proinsulin mediated by RILP reduces insulin secretion [50], whereas limiting this degradative pathway preserves hormone availability. SINGD further demonstrates that excessive lysosomal degradation can contribute to insulin depletion and β-cell dysfunction during metabolic stress.

These observations suggest that therapeutic inhibition of crinophagy may not be uniformly beneficial. While reducing crinophagic processing could potentially decrease the generation of HIPs and other neoepitopes implicated in T1D, prolonged suppression of physiological granule turnover might impair SG quality control, alter insulin availability, and compromise β-cell homeostasis [62,68]. At present, direct evidence linking selective crinophagy inhibition to proteotoxic stress or activation of canonical endoplasmic reticulum stress pathways remains limited. Additionally, impaired clearance of aged or oxidatively damaged granule contents could theoretically increase intracellular oxidative burden, further compromising β-cell viability and insulin secretory competence, though this risk also remains to be directly demonstrated in the context of crinophagy inhibition. Consequently, such outcomes should be regarded as plausible but unproven risks.

Future therapeutic strategies may therefore require selective modulation rather than complete inhibition of crinophagy. Approaches that specifically target antigen-generating pathways while preserving physiological granule turnover could provide a more favorable balance between immune benefit and maintenance of β-cell function. Defining the molecular determinants that distinguish homeostatic crinophagy from pathogenic antigen-generating pathways will be an important prerequisite for the rational development of crinophagy-directed therapies.

### 4.5. Type 2 Diabetes: Stress-Induced Nascent Granule Degradation and β-Cell Failure

Whereas the pathogenic contribution of crinophagy in type 1 diabetes is often framed around neoepitope generation, lysosomal granule degradation pathways in type 2 diabetes are more closely linked to loss of granule mass and secretory capacity. A noticeable example is SINGD, a pathway in which newly formed granules are diverted from the biosynthetic–secretory route to lysosomes under conditions such as glucolipotoxic stress (Figure 5) [18]. Unlike the mature-granule crinophagy discussed in T1D, SINGD targets newly synthesized nascent granules and contributes primarily to β-cell dysfunction through insulin depletion rather than neoepitope generation.

## 5. Consequences: Insulin Depletion and Impaired Cellular Maintenance

The sustained lysosomal degradation of SGs under metabolic stress has consequences that extend beyond insulin depletion. To understand this, it is critical to examine how SG degradation biochemically signals to the rest of the cell. Goginashvili et al. demonstrated in INS-1 rat insulinoma cells that amino acids released from lysosomal SG degradation during SINGD activate mTORC1 at the lysosomal surface, which in turn suppresses macroautophagy initiation [51]. These findings support a model in which increased lysosomal SG degradation could contribute to a self-reinforcing cycle whereby amino acid release activates mTORC1, suppresses macroautophagy, and thereby exacerbates β-cell dysfunction under chronic glucolipotoxic stress [18].

The degree to which individual SG degradation routes can substitute for one another depends on which cellular machinery is disrupted. In Rab3A-deficient β-cells, where regulated exocytosis is impaired and SGs accumulate, enhanced lysosomal degradation through crinophagy- and autophagy-related pathways partially compensates, limiting excessive cytoplasmic SG buildup [5]. This indicates that when the secretory route is blocked, lysosomal degradation pathways can be upregulated to absorb the excess granule load. In contrast, genetic perturbations that directly disable core crinophagy machinery, including RAB27A, Munc13-4, and VAMP2 in the BON cell model, impair SG–lysosome fusion and result in SG accumulation that is not adequately compensated by alternative degradative routes [21]. Together, these contrasting observations define the limits of pathway redundancy in β-cell SG homeostasis: alternative lysosomal routes can partially buffer against secretory failure, but loss of dedicated crinophagy machinery in the BON cell model is not readily compensated by alternative degradative routes. This underscores a central principle: balanced lysosomal routing is required for SG homeostasis, and both insufficient clearance, leading to SG accumulation, and oxidative damage and excessive degradation, reducing the functional insulin pool, can be detrimental depending on metabolic context [5,21].

## 6. Limitations and Outstanding Challenges

Despite substantial progress, several important limitations constrain current understanding of β-cell crinophagy and its contribution to diabetes pathogenesis (Table 2).

First, much of the mechanistic framework discussed in this review is derived from experimental systems rather than human pancreatic β-cells. Key insights into crinophagic trafficking have emerged from BON human neuroendocrine cells [21], *Drosophila* secretory tissues [48], INS-1 and MIN6 β-cell lines [18,46,51], and the NOD mouse autoimmune model [22], each of which provides valuable mechanistic insights but only partially reflects the physiological and immunological complexity of human pancreatic β-cells.

Second, direct evidence linking crinosome formation to hybrid insulin peptide (HIP) generation and subsequent antigen presentation in human disease remains limited. While HIPs and HIP-reactive CD4^+^ T cells have been detected in human islets and peripheral blood, the complete crinosome → HIP → antigen presentation → T-cell activation pathway has not yet been demonstrated in vivo in humans [67]. Furthermore, crinosomes have not yet been directly isolated and molecularly characterized from human diabetic islets. Consequently, several aspects of this model should currently be regarded as biologically plausible rather than definitively established.

Third, the field lacks robust biomarkers for monitoring crinophagic activity in clinical samples. No validated circulating biomarkers, imaging approaches, or crinosome-specific molecular signatures currently permit direct assessment of crinophagic flux in vivo. This limitation complicates efforts to determine whether crinophagy is altered during diabetes progression or in response to therapeutic intervention.

Fourth, the molecular distinction between constitutive crinophagy of mSGs, SINGD, and other lysosome-dependent granule degradation pathways remains incompletely resolved. Greater mechanistic clarity, together with more consistent terminology across the field, will be required to define the specific contribution of each pathway to β-cell physiology and disease.

Finally, therapeutic targeting of crinophagy remains largely speculative. Although experimental studies suggest that modulation of crinophagic pathways may influence autoimmune epitope generation or secretory granule turnover, the long-term consequences of altering this fundamental quality-control mechanism remain unknown. Notably, a clinical trial of hydroxychloroquine in stage 1 T1D patients did not delay progression to stage 2 disease despite reducing autoantibody titers, highlighting the complexity of translating lysosomal or crinophagy-targeted interventions into clinical benefit. Future studies in primary human islets, stem-cell-derived β-like cells, and humanized disease models will be essential for establishing both mechanistic validity and translational feasibility.

## 7. Future Directions and Outstanding Priorities

Despite substantial advances in our understanding of β-cell crinophagy, several fundamental questions remain unresolved. Most notably, key mechanistic pathways have yet to be validated in primary human β-cells, reliable biomarkers of crinophagic activity are lacking, and experimental systems capable of visualizing crinosome dynamics in real time remain underdeveloped. Addressing these gaps will be essential to determine whether crinophagy contributes causally to diabetes pathogenesis and whether it represents a tractable therapeutic target. We propose five immediate priorities for future investigation.

### 7.1. Validation in Human β-Cells and Islets

Much of the current mechanistic framework derives from BON cells, rodent β-cell lines, and animal models. Direct validation of the proposed crinophagy machinery—including RAB27A–Munc13-4 interactions, SNARE-mediated fusion events, and PLEKHM1–HOPS-dependent tethering—in primary human islets represents a major priority. Such studies will be essential to establish the extent to which current models accurately reflect human β-cell biology.

### 7.2. Stem-Cell-Derived β-like Cell Platforms

Human stem-cell-derived β-like cells offer a scalable system for mechanistic studies and live-cell imaging. Coupling fluorescent reporters of secretory granules and lysosomes with advanced imaging approaches could enable direct visualization of crinosome formation, granule aging, and crinophagic flux under physiological and diabetogenic conditions.

### 7.3. Crinosome Proteomics and Immunopeptidomics

Isolation and molecular characterization of crinosomes from human islets would provide a critical resource for defining their protein, peptide, and neoepitope composition. Comparative analyses across human islets, stem-cell-derived β-like cells, and established animal models may clarify the translational relevance of currently proposed mechanisms, including HIP generation.

### 7.4. Biomarker Discovery

The absence of validated biomarkers currently limits assessment of crinophagic activity in vivo. Identification of circulating HIP species, lysosome-associated granule components, or other crinosome-derived signatures could enable longitudinal monitoring of β-cell stress and granule turnover during diabetes progression and therapeutic intervention.

### 7.5. Genetic Models of Selective Crinophagy Modulation

The development of inducible β-cell-specific models targeting candidate crinophagy regulators will be important for establishing causality in both autoimmune and metabolic diabetes. Such approaches may help distinguish physiological granule turnover from pathological degradation pathways while avoiding the systemic effects associated with pharmacological interventions.

Overall, these priorities converge on a central goal: establishing whether crinophagy is merely associated with β-cell dysfunction or represents a mechanistically actionable pathway in diabetes pathogenesis. Progress in this area will require integrating human islet biology, advanced imaging, proteomics, and genetically precise model systems to bridge the current gap between mechanistic insight and therapeutic translation.

## 8. Conclusions

Crinophagy occupies a unique position at the intersection of secretory biology, metabolic homeostasis, and immune tolerance. The emerging picture is one of a single degradative pathway serving two distinct but interconnected functions: physiological quality control, through the selective removal of aged or dysfunctional insulin granules to maintain secretory competence, and immunological consequence, through the generation of crinosome-associated neoepitopes that may expand the peripheral antigen repertoire and potentiate autoreactive CD4^+^ T-cell responses in type 1 diabetes. Although many mechanistic details remain unresolved, current evidence suggests that the molecular machinery proposed to mediate homeostatic granule turnover may also influence crinosome abundance and composition and, consequently, the generation of hybrid insulin peptides and other modified antigens. Conversely, excessive lysosomal degradation of insulin granules, as observed in SINGD, may contribute to β-cell dysfunction in T2D by depleting the secretory granule pool, underscoring the context-dependent consequences of granule degradation pathways. This conceptual framework highlights how perturbations of crinophagy could carry both metabolic and immunological consequences, a duality that should be considered when evaluating the therapeutic potential of this pathway.

Despite these advances, fundamental questions remain unresolved. How are aged or dysfunctional secretory granules selectively marked for lysosomal entry? What molecular decision points determine whether granules undergo constitutive crinophagy, undergo stress-induced nascent granule degradation (SINGD), or are spared in favor of secretion? How do these degradation pathways intersect with β-cell identity programs, dedifferentiation, and survival under chronic metabolic stress? Most critically, can crinophagy be therapeutically modulated to preserve β-cell function or limit autoimmunity without compromising essential quality-control mechanisms?

Addressing these questions will require integrated efforts across cell biology, immunology, genetics, and translational research. Emerging approaches, including high-resolution live-cell imaging, single-cell and spatial omics, human stem-cell-derived islet models, and systems-level analyses, now provide the tools needed to interrogate crinophagy with unprecedented precision in physiologically relevant contexts.

More broadly, insights from crinophagy research are likely to extend beyond pancreatic β-cells. Secretory cells across endocrine, neuroendocrine, and exocrine systems face a shared challenge: balancing sustained biosynthesis with regulated secretion while avoiding toxic accumulation of obsolete cargo. Crinophagy offers a paradigm for how this balance can be achieved through selective, organelle-level degradation.

The field of crinophagy research thus stands at a pivotal moment. With molecular mechanisms in hand, human-relevant model systems emerging, and a conceptual framework that links organelle biology to metabolic and immune disease, the opportunity now exists to translate decades of descriptive and mechanistic work into therapeutic strategies. The trajectory from morphological curiosity to molecular medicine is no longer speculative; it is actively unfolding.

## 9. Literature Search Strategy

This narrative review was informed by literature searches conducted using PubMed, Web of Science, and Google Scholar. Search terms included “crinophagy”, “secretory granule degradation”, “insulin granule turnover”, “hybrid insulin peptides”, “SINGD”, “crinosome”, “β-cell lysosomal pathway”, “crinophagy and type 1 diabetes”, “type 2 diabetes” and related keywords. Studies published between 1966 and early 2026 were considered. Priority was given to primary research articles, seminal studies establishing key concepts, and recent investigations providing mechanistic or translational insight. Where available, findings from human islets and human β-cells were preferentially cited and explicitly distinguished from evidence derived from non-β-cell models, rodent β-cell lines, or animal studies. Foundational reviews were included to provide historical and conceptual context.

## Figures and Tables

**Table 1:** Comparison of lysosomal degradation pathways for insulin secretory granules in pancreatic β-cells.

Feature	Macroautophagy	Microautophagy	Crinophagy
Membrane mechanism	Double-membrane autophagosome sequesters SG; outer membrane fuses with lysosome; inner membrane and SG cargo are delivered intact into the lysosomal lumen	Lysosomal or late endosomal limiting membrane directly invaginates or protrudes to engulf SG material; no intermediate double-membrane structure	Direct fusion of the SG single limiting membrane with the lysosomal single limiting membrane; no intermediate autophagosome
SG membrane fate	Degraded—both the SG membrane and the autophagosomal inner membrane are broken down within the autolysosome	Degraded—SG membrane is incorporated into the multivesicular body and degraded within the lumen	Preserved—SG membrane merges with the lysosomal membrane; components potentially recycled
SG content fate	Complete degradation of both cargo and membrane components	Complete degradation of both cargo and membrane components	Selective degradation of luminal cargo (insulin, C-peptide, prohormone); membrane components potentially spared
ATG protein dependence	Yes—ATG5, ATG7, ULK1, LC3 lipidation, and VPS34–PI3K complex are required	Variable—ATG-dependent and ATG-independent forms have been described; not fully characterized in β-cells	No—ATG-independent; proceeds independently of canonical autophagy machinery
Key molecular machinery	ULK1–ATG13–FIP200 initiation complex; VPS34–PI3K; LC3–PE conjugation; HOPS/PLEKHM1 tethering; SNARE fusion: STX17–SNAP29–VAMP8 (canonical) or YKT6–SNAP29–STX7 (alternative)	Not fully characterized in β-cells; RAB7 and ESCRT machinery implicated; STX7-containing SNARE complexes proposed in some contexts	RAB27A, Munc13-4 (SG-associated); VAMP2–STX7–STX8–VTI1B SNARE (mature mSG fusion) or VAMP4–STX7–STX8–VTI1B (iSG/SINGD); HOPS/PLEKHM1 tethering (lysosome) [characterized in BON cell model]
Preferred SG substrate	Both iSG and mSG; non-selective cargo capture	Preferentially mSG; mechanism not fully defined in β-cells	Preferentially aged mSG under basal conditions; nascent iSG under glucolipotoxic stress (SINGD)
Physiological context	ER stress, oxidative damage, prolonged nutrient deprivation, high-fat diet	Impaired exocytosis (e.g., RAB3A deficiency); constitutive membrane remodeling	Constitutive homeostatic turnover; upregulated when insulin biosynthesis exceeds secretion
Metabolic regulation	Strongly induced by nutrient deprivation and mTORC1 inhibition; AMPK-dependent activation	Context-dependent; incompletely defined in β-cells	Constitutive; SINGD branch further upregulated by glucolipotoxic stress via reduced PKD1 activity; mTORC1 activated downstream by lysosomal amino acid release
Age selectivity of SG targeting	Non-selective—random cargo capture regardless of granule age	Non-selective/unknown	Preferential degradation of aged mSG (proposed RAB27A/RAB3A ratio model); nascent iSG targeted under stress
Hybrid organelle formed	Autolysosome (double-membrane autophagosome fused with lysosome)	Multivesicular body/late endosome with internalized SG-derived vesicles	Crinosome—hybrid SG–lysosome organelle with acidic, protease-rich lumen
Contribution to basal SG turnover	Minor under non-stress conditions; primarily a stress-response pathway	Minor; predominantly observed under conditions of impaired exocytosis	Major proposed pathway for constitutive basal SG turnover in unstressed β-cells
Disease relevance	β-cell dysfunction in Atg7 and Atg5 conditional KO models (glucose intolerance, protein aggregate accumulation); general stress response in T2D	SG accumulation in exocytosis-deficient models (e.g., RAB3A KO); disease relevance not fully established	Mature mSG crinophagy: HIP generation → peripheral–thymic mismatch → T1D autoimmunity; nascent iSG SINGD: insulin pool depletion → progressive T2D β-cell failure

Abbreviations: iSG, immature secretory granule; mSG, mature secretory granule; SG, secretory granule; SINGD, stress-induced nascent granule degradation; HIP, hybrid insulin peptide; KO, knockout; ATG, autophagy-related. The crinophagy molecular machinery listed is characterized primarily in BON neuroendocrine cells and requires direct validation in primary pancreatic β-cells. The right-pointing arrow (→) denotes ‘leads to’ or a sequential progression of events.

**Table 2:** Translation context and knowledge gaps for key β-cell degradative mechanisms.

Vesicular/Degradative Mechanism	Validated in Human Tissue?	Primary Experimental Model(s)	Ref.	Major Knowledge Gap
iSG to mSG Maturation Switch (Proteomic/lipidomic aging, RAB3A association with young SGs)	No (Currently Inferred)/Partial	INS-1 cells	[21,37]	No validation in primary human β-cells; proteomic/lipidomic aging data confined to rodent insulinoma lines; RAB3A association not tested in human islets
Crinosome Formation (mSG fusion with lysosomes via SG proteins Rab27A, Munc13-4, VAMP2 and lysosomal proteins STX7, STX8, VTI1B, PLEKHM1, HOPS)	Partial/Inferred *	BON cells, primary mouse β-cells—Munc13-4 KO	[21]	Direct ultrastructural and molecular characterization in human islets absent; Munc13-4 KO findings not replicated in human islet systems; BON cell data requires human β-cell confirmation
Hybrid Insulin Peptide (HIP) Generation (Transpeptidation/reverse proteolysis mediated by Cathepsins L, D, S, B)	Yes (Validated via CD4^+^ T-cell responses in human islet-infiltrating T- cells, PBMCs, and proteomics of enriched insulin granules)	Biochemically purified-enzyme digests, lysosomal assays, enriched insulin granules, CatL-deficient islets, NOD mice, Human PBMCs	[60,62,63,65,66]	Definitive sub-compartmental localization of transpeptidation within crinosomes vs. other lysosomal compartments unresolved; cathepsin isoform hierarchy in human β-cells unclear
SINGD (Lysosomal degradation of nascent granules controlled by PKD to suppress autophagy)	Yes (Functional effects on insulin secretion validated via tat-beclin1 in ex vivo fasted human islets)	INS1 cells, primary murine islets, LC3B-GFP mice, p38δ knockout mice, human islets	[18,51,58]	Clinical biomarkers for SINGD activity in vivo lacking; longitudinal validation in diabetic human islets needed; PKD isoform specificity in human β-cells undefined
TFEB-mediated lysosomal biogenesis and macroautophagy	Yes (Decreased TFEB nuclear staining associated with human pancreatitis)	Acinar cell-specific *tfeb* KO mice, *tfeb* and tfe3 DKO mice, GFP-LC3 transgenic mice	[57]	All mechanistic data from acinar/exocrine models; β-cell-specific regulation of crinophagy via TFEB uncharacterized; relevance to insulin granule turnover in human islets unestablished

INS-1—rat insulinoma; BON—neuroendocrine human cell line; PBMC—Peripheral Blood Mononuclear Cell; SINGD—Starvation-Induced Nascent Granule Degradation; iSG—immature secretory granule; mSG—mature secretory granule; SG—secretory granule; TFEB, transcription factor EB; KO—knockout; DKO—double knockout. * Partial/Inferred: crinophagy observed via electron microscopy and immunofluorescence in rodent β-cell models; direct human islet confirmation absent.

## Data Availability

No new data were created or analyzed in this study. Data sharing is not applicable to this article.

## Abbreviations

The following abbreviations are used in this manuscript:

SGs	Secretory Granules
iSGs	Immature Secretory Granules
mSGs	Mature Secretory Granules
SINGD	Stress-Induced Nascent Granule Degradation
SNARE	Soluble NSF Attachment Protein Receptor
HIPs	Hybrid Insulin Peptides
TGN	Trans-Golgi Network
HOPS	Homotypic Fusion and Protein Sorting Complex
PLEKHM1	Pleckstrin Homology and RUN Domain-Containing M1
VAMP	Vesicle-Associated Membrane Protein
TRPML1	Transient Receptor Potential Mucolipin 1
ROS	Reactive Oxygen Species
NOX2	NADPH Oxidase
V-ATPase	Vacuolar-Type H^+^-ATPase
